# In-house designed Multiplex Real-Time PCR Assay Developed for Epstein - Barr virus and Cytomegalovirus DNA detection in Pooled Blood Samples from Iraqi blood donors

**DOI:** 10.12688/f1000research.173833.2

**Published:** 2026-06-25

**Authors:** Ghinwa S. Majid, Ahmed S. Abdulamir, Iman M. Aufi

**Affiliations:** 1 Biology, Alnahrain University, College of sciences, Baghdad, 10001, Iraq; 2 College of Medicine, Al-Nahrain University, Baghdad, Iraq; 3 Central Public Health Laboratory, Baghdad, Iraq

**Keywords:** CMV, EBV, multiplex PCR, blood donors, cost-effectiveness, blood transfusion. viral detection, In-house designed

## Abstract

**Background:**

The problem of transmissible human herpesvirus infections through blood donation has not been completely resolved by serological testing due to issues such as delayed in seroconversion or false-negative results. This study aimed to develop a cost-effective, In-house designed multiplex real-time PCR assay for the simultaneous detection of Epstein–Barr virus (EBV) and cytomegalovirus (CMV) DNA in pooled blood donor samples.

**Methods:**

A total of 1000 whole-blood samples were collected from apparently healthy Iraqi blood donors and pooled into mini pools 10(MP10) i.e. 100 MP10 tested by a commercial CMV and EBV multiplex- qPCR detection kit; then retested by In-house designed multiplex qPCR. Fifteen patients who were positive for CMV and fifteen for EBV were considered as positive groups and fifteen healthy individuals as negative groups used to estimate the validity and feasibility of the In-house designed kit.

**Results:**

Blood donors who tested by the commercial kit were 42% MP10 positive for EBV and 4% positive for CMV while the In-house designed multiplex qPCR results were 40% and 6% for EBV and CMV, respectively. The specificity and sensitivity of the In-house designed kit for the detection of CMV were 97.92% (95% CI 92.6 to 99.7), 100% (95% CI 39.7 to 100), for EBV were 100% (95% CI 93.84 to100.00) and 95.24 % (95% CI 83.84 to 99.42), respectively.

**Conclusion:**

The rate of EBV was high within Iraqi blood donors while the CMV was moderately low and the In-house designed multiplex qPCR was sensitive and specific for CMV and EBV DNA detection at a much lower cost than that of the commercial, about 10% of the commercial cost. Such a cost-effective and sensitive test is crucial in reducing blood transfusion-associated diseases.

## Introduction

Therapy through blood transfusion is still considered one of the safest and most effective methods for managing cases with significant morbidity or mortality, and it is widely used in medicine, particularly for haematological and other systemic diseases ( Busch *et al.*, 2019). However, transfusion-transmissible infections remain among the most serious problems associated with blood transfusion ( Mengjiao *et al*., 2024). Epstein–Barr virus (EBV) and cytomegalovirus (CMV) are human herpesviruses that establish lifelong latency. They can compromise the safety of blood transfusion, particularly in immunocompromised patients, and are among the most common infectious agents linked to post-transfusion complications, especially in solid organ transplant recipients with a high risk of opportunistic infections ( Bunchorntavakul & Reddy, 2020; Wada *et al*., 2007). Other high-risk groups include seronegative recipients of bone marrow transplants, seronegative patients with haematological malignancies, pregnant women, and premature infants born to seronegative mothers ( de Melo Silva *et al.*, 2020).

Therefore, qualitative or quantitative detection of these viruses could help prevent diseases or complications arising from the transfusion of blood containing reactivated or recently acquired EBV or CMV infections ( Gunson *et al*., 2009). The implementation of multiplex PCR assays enables rapid and precise detection of viral infections through nucleic acid amplification techniques (NAT). This approach offers a convenient, time-efficient, and cost-effective screening tool for both clinical and research laboratories ( Luzius *et al*., 2025; Markoulatos *et al.*, 2002). Furthermore, the use of pooled samples in multiplex PCR assays has proven highly efficient in reducing testing costs ( Grobe *et al*., 2021; Smatti *et al.*, 2017; Yamkasem *et al.*, 2025). Detection of EBV and CMV in whole blood has been shown to be more sensitive, as the DNA levels of them are generally higher in whole blood samples from the same patient ( Fukuda *et al*., 2024; Rzepka *et al*., 2022). Traditionally, EBV and CMV reactivation has been diagnosed using serological methods; however, serological tests are less accurate and may yield false-negative results due to their inability to detect viral antigens or antibodies in samples with low viral loads, particularly in newly infected or immunosuppressed individuals ( Nemr & Nashwa, 2024; Smatti *et al., *2017). To the best of our knowledge, no previous studies have investigated circulating EBV and CMV DNA levels in the blood of Iraqi blood donors using whole blood samples. Therefore, this study aimed to estimate the prevalence of EBV and CMV DNA among apparently healthy Iraqi blood donors by analyzing pooled whole blood samples using a multiplex qPCR assay. Moreover, it sought to develop a cost-effective, In-house designed multiplex qPCR kit for detecting EBV and CMV DNA in blood donors and to compare the performance of this In-house kit with that of a commercially available assay targeting the same viruses ( Sacace Biotechnologies, 2016).

## Materials and methods

### Study population

A developmental cross-sectional study was designed for a total of one thousand healthy Iraqi blood donors who donated blood through the National Iraqi Blood Bank in Baghdad City from July 2018 to January 2019, and all of them were seronegative for HBV, HCV, and HIV according to the routine screening tests conducted for viral detection within the Iraqi Blood Bank. This study was implemented in accordance with the Declaration of Helsinki ( https://www.wma.net/policies-post/wmadeclaration-of-helsinki). Ethical approval was obtained from the Iraqi Ministry of Health/national center for training and human development No. 1009 in 24/6/2018 and from Institutional Review committee (IRB) in College of Medicine-university of AlNahrain No. 577 in 16/4/2018 to get this approval. All patients and blood donors provided written informed consent before participation. The consent process included a detailed explanation of the study objectives, the sample collection procedures, and the potential risks and benefits. Participants were informed that their data would remain confidential and anonymized. Written consent forms were signed by each participant and archived according to institutional requirements.

### Samples, controls and pooling of samples

Sets of pooled whole blood samples prepared for validation consisting of 5, 10 and 15 samples, they were containing one CMV-positive whole-blood sample and one EBV-positive whole-blood sample, while the rest of pooled samples were negative for both viruses. The results revealed that the 5-sample and 10-sample pools yielded promising outcomes, whereas the 15-sample pool did not.

About 150 μl was pipetted from each of ten whole blood samples (individual samples) and then mixed, i.e., pooled into one set of 10 samples with a total volume of 1.5 ml of pooled whole blood, thereby reducing the number of samples to 100 mini-pooled (MP10) whole blood samples from blood donors. A positive control was also included, consisting of fourteen patients confirmed to have CMV infection through serology (IgM+) and nucleic acid tests (NAT), or NAT tests only, and thirteen patients confirmed to have EBV infection through serology using viral capsid antigen (VCA IgM+) and NAT, or NAT tests only. Furthermore, a negative control group of fifteen whole blood samples from healthy individuals was included as negative controls for CMV and EBV, based on NAT test results only.

Then all samples were stored frozen at −80 °C, in accordance with the manufacturer’s instructions, until processing.

### DNA extraction and amplification by commercial kit

DNA extraction of the viruses was done from 200 μl MP10 whole blood of blood donors, positive and negative control groups by QIAamp ^®^ DNA Mini and Blood Mini 250 (Lot no. 151043756 Qiagen/Germany). Then, eluted DNA samples were stored at −20°C. CMV/EBV/HHV6 Quant Real-TM kit (Lot no. 27H181705 sacace/Italy) was used for the detection and differentiation of Cytomegalovirus (CMV) and Epstein Barr Virus (EBV) simultaneously by multiplex qualitative Real-time PCR using 10 μl of extracted DNA as manufacturer’s instructions.

### Development of In-house designed multiplex qPCR

Primers and Taqman probes were designed based on conserved regions of the genome for each virus, CMV DNA polymerase gene or UL54 (152pb) and EBV DNA polymerase gene (150pb), and B-actin gene used as an internal control. These were retrieved from Genbank from NCBI ( www.ncbi.nlm.nih.gov), then the primer and probes were designed for these genes in NCBI ( www.ncbi.nlm.nih.blast). Different fluorochromes were used for Taq-man probe labeling, the EBV probe was labeled with Rox fluorescent dye at the 5′ and quenched with Black-Hole-Quencher2 at the **3′** (BHQ2); while CMV probe was labeled with FAM fluorescent dye and quenched with BHQ1 ( Table 1). After that to check the specificity of designed primers and probes for target genes, they were queried against the GenBank nucleotide database using BLASTN. All primers and probes were manufactured by IDT company.

**Table 1.** In-house designed primers and Taq-man probes.

VIRUS NAME	
CMV-F	5-ACTGTAGCCGTGTTCTGTG-3
CMV-R	5-CACCTACGATCAGACGGACG-3
CMV-Probe	FAM"TGACGATAGCGCGGCGACAC"BHQ1
EBV-F	5"GAAAAGCAGAGCTCCCCCA"3
EBV-R	5-CGGCCCTTCCAAGAGTCATT-3
EBV-Probe	Rox"GGGGACCCTGCCTTCACGGA"BHQ2
B-actin-F	CGTGCTCGATGGGGTACTTC
B-actin-R	GCTCAGGGCTTCTTGTCCTTT
B-actin-probe	HEX-TGGGCCTCGTCGCCCACATA-BHQ1

CMV, Cytomegalo viruse; EBV, Epstein Bar viruse.

### DNA amplification

Gradient PCR was performed and 63°C was chosen as a suitable annealing temperature for multiplex PCR reaction. Then DNA from two positive whole blood samples for CMV and EBV was mixed, and the 20 μl qPCR reaction was prepared by adding 10 μl of GoTaq ^®^ probe qPCR Master Mixes (Promega/USA), 0.5 μl of 10 μm primers mix, 0.5 μl of 10 μm probes mix, 0.3 μl of extracted human DNA, 0.7 μl of free nuclease DW and 8 μl of DNA template were added. The qPCR reactions were loaded in MicroAmpR fast Reaction tubes (8 tubes/strips) (Appliedbiosystem/USA) and placed in a Real-time fast 7500 thermocycler (Appliedbiosystem/USA). The thermoprofile of the multiplex PCR reaction was 1 cycle 2 mins at 95°C, 40 cycles for 10 secs at 95°C, 40 secs at 63°C, 15 sec at 72°C, and 1 cycle for 1 min at 72°C.

### Primer efficiency and determination the detection limit

Tenfold serial dilution was done through human negative whole blood and the viral concentration spanning (10 ^3^, 10 ^2^, 10) copies/10 ^5^ cells, and then tested in triplicate. The standard curves were plotted resulting from Ct value versus log of viral copy numbers.

### Statistical analysis

SPSS version 16.0.0, Microsoft Excel 2010, and GraphPad Prism version 7.04 were used for data processing. The Student’s *t*-test and ANOVA were applied for parametric data, while the Mann–Whitney test was used for non-parametric data to determine the significance of differences in means, taking into account whether the variables under analysis shared equal or different variances. In the current study, the coefficient of variation (CV) defined as the standard deviation divided by the mean and multiplied by 100 was used to obtain a percentage. For intra-assay CV, the average CV across all replicates of a single sample within one assay run was calculated, followed by calculating the overall average of these CVs. Conversely, for inter-assay CV, the mean of each sample across multiple runs was calculated, and the CVs derived from these means were then averaged.

## Results

### 1- Amplification of commercial NAT kit

**Figure 1.**
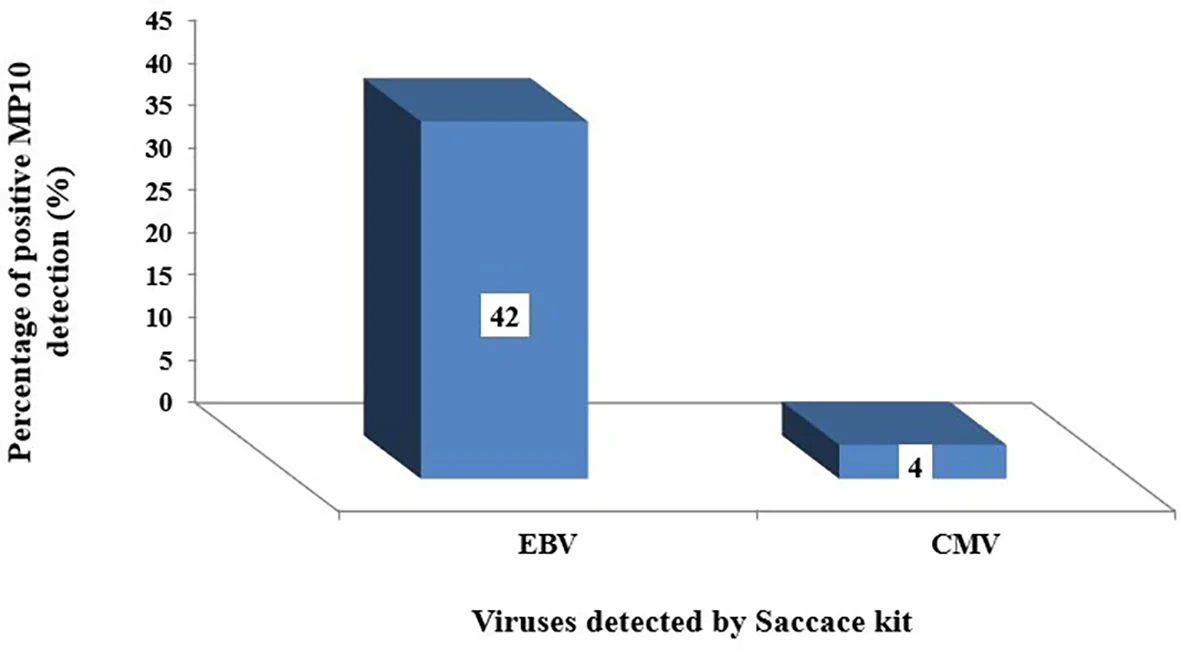
Bar chart showing the proportion of MP10-positive whole blood samples testing positive for EBV and CMV using the commercial multiplex qPCR kit. The EBV-positive rate reached (42%), whereas CMV showed a lower positivity rate of (4%).

The results of the commercial NAT assay using multiplex Real-Time qPCR revealed that 4 out of 100 (4%) mini-pools of whole blood were CMV-positive, while 42 out of 100 (42%) mini-pools were EBV-positive among Iraqi blood donors ( Figure 1). NAT-positive pools were resolved by testing the individual donor samples using multiplex real-time PCR. The positive pools demonstrated that each EBV-positive MP10 contained, on average, three positive individual samples (i.e., out of 10 samples were positive for EBV), whereas each CMV-positive MP10 included one individual positive sample. In addition, two mini-pools tested positive for both EBV and CMV.

### 2- The In-house designed multiplex qPCR

**Figure 2.**
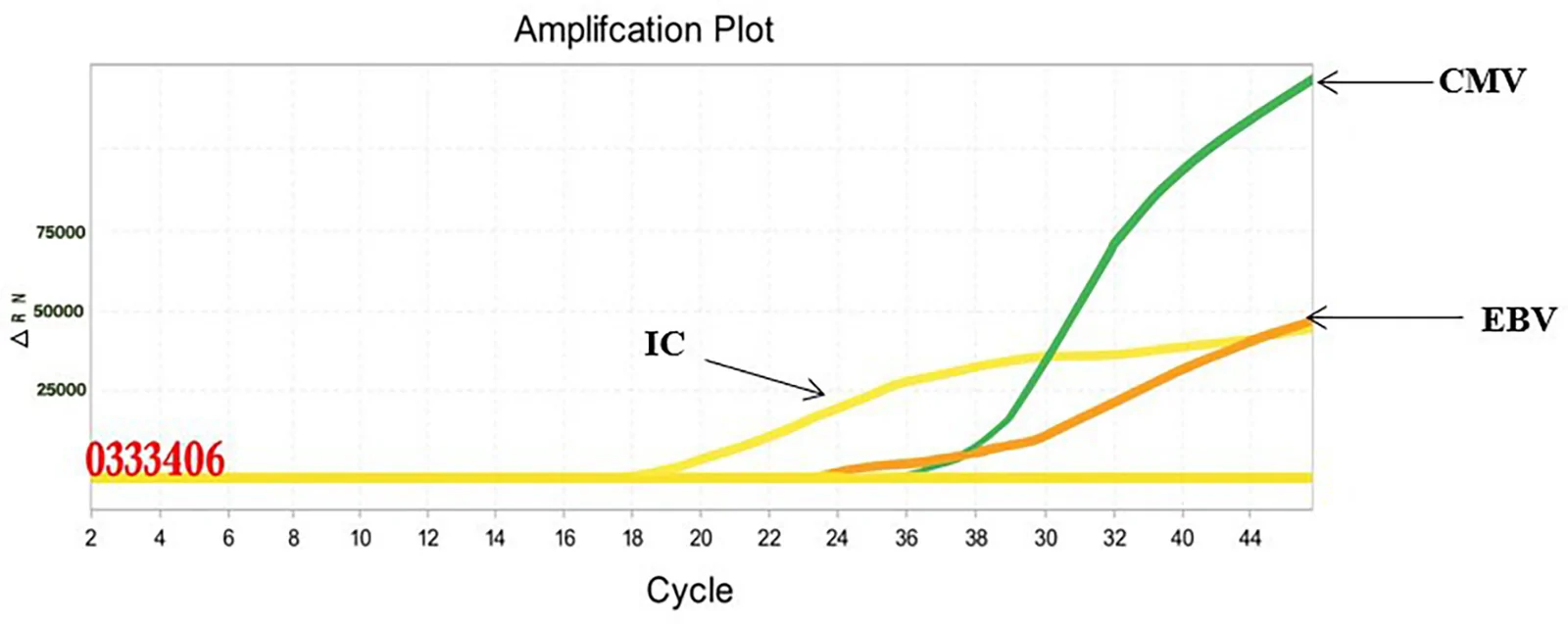
Amplification curves of multiplex qPCR showing simultaneous detection of CMV, EBV, and the internal control (IC). The CMV target is represented by the green curve, the EBV target by the orange curve, and the IC by the yellow curve. Both viral targets, along with the internal control DNA, were successfully amplified within the same reaction mixture, confirming assay specificity and proper reaction performance.

The positive and negative controls used for the estimation of the validity and feasibility of In-house designed multiplex qPCR are shown in Figure 2

.

**Primers efficiency and limit of detection (LOD)**

**Figure 3.**
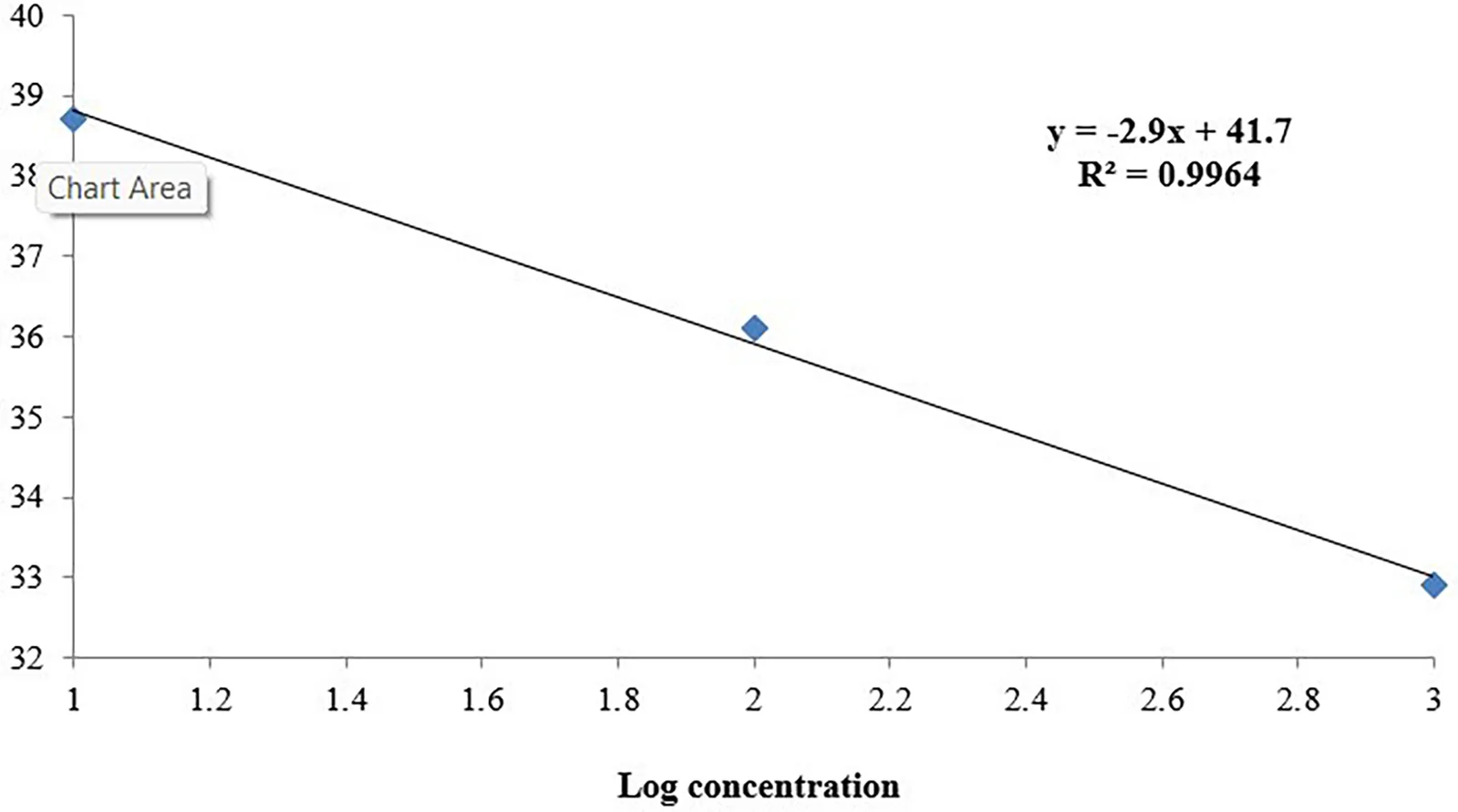
Standard curve generated from serial dilutions of a single CMV-positive sample. The curve shows the linear relationship between the log of the template concentration and the corresponding Ct values, with a regression equation of y = −2.9x+41.7, and a coefficient of determination R ^2^ = 0.9964. The calculated amplification efficiency for CMV was 110%.

**Figure 4.**
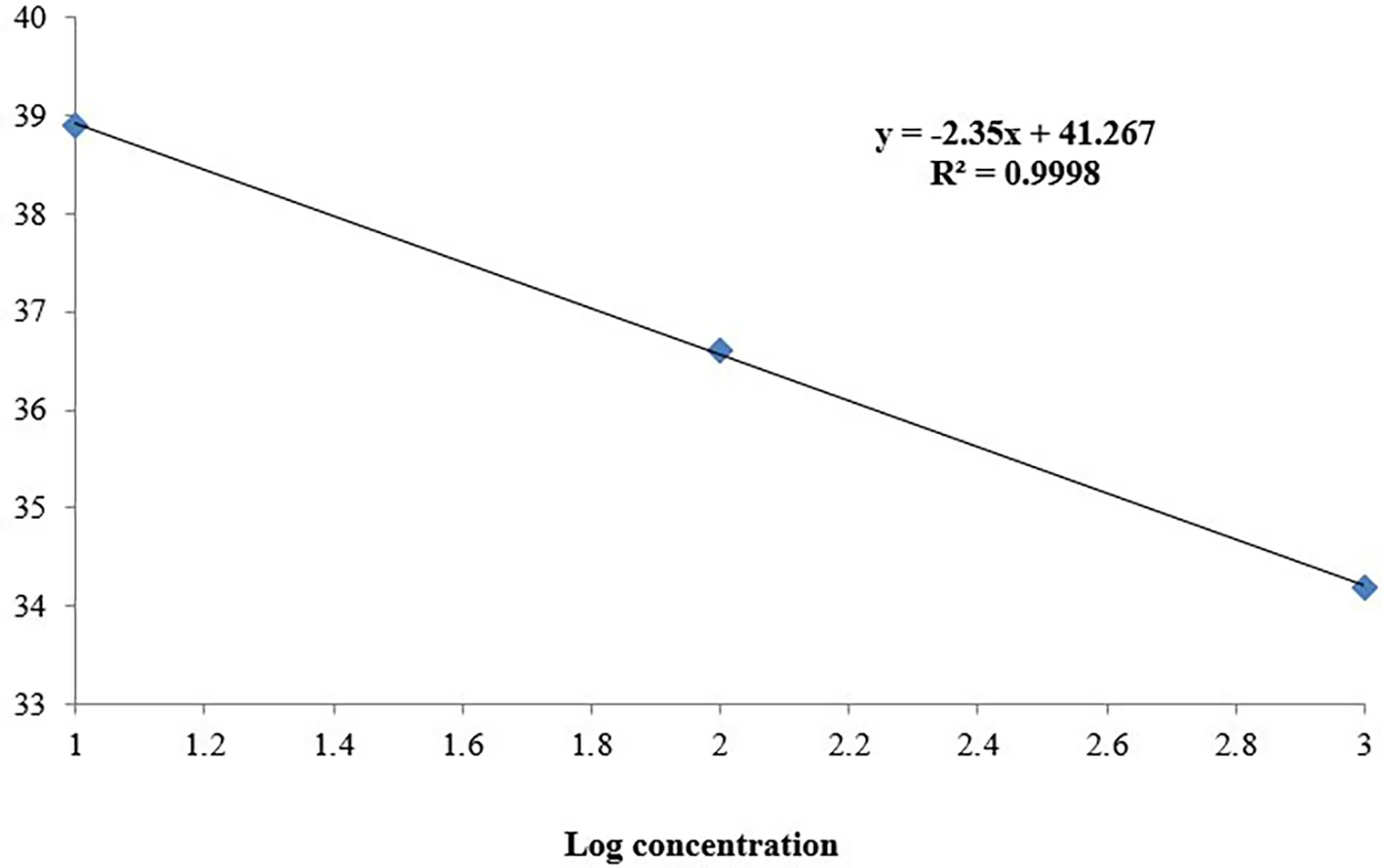
Standard curve derived from serial dilutions of the positive EBV sample, illustrating the linear correlation between the logarithm of template concentration and the corresponding Ct values. The regression equation was y =−2.35x+41.267, with a coefficient of determination R ^2^ = 0.9998, indicating excellent linearity of the assay. The amplification efficiency calculated from the slope was 100% consistent with optimal qPCR performance.

The viral load of CMV- and EBV-positive whole blood samples (10 ^3^ copies/10 ^5^ cells) was used to determine the detection limit of the In-house-designed primers and probes for CMV and EBV. Tenfold serial dilutions were prepared in human CMV/EBV-negative whole blood, with concentrations of 10 ^3^, 10 ^2^, and 10 ^1^ copies per 10 ^5^ cells. Triplicates of each dilution were tested using the In-house-designed multiplex qPCR. The results revealed that the amplification efficiency for CMV and EBV PCR assays was 110% and 100%, respectively, as shown in Figures 3 and 4. The limit of detection (LOD) is presented in Tables 2 and 3.

**Table 2.** Limit of detection (LOD) of EBV DNA by In-house designed multiplex qPCR.

EBV LOD (one target)	CT values		
TRIPLICATE 1000 copies/10 ^5^ CELLS	33.6	34	34.1
TRIPLICATE 100 copies/10 ^5^ CELLS	36.9	36	36.3
TRIPLICATE 10 copies/10 ^5^ CELLS	38.5	38.9	38.3
EBV **LOD =10** copies/10 ^5^ CELLS, **CT value ranged (33-39)± 2**			

**Table 3.** Limit of detection (LOD) of CMV by In-house designed multiplex qPCR.

CMV LOD (one target)	CT values		
TRIPLICATE 1000 copies/10 ^5^ CELLS	32.5	32.8	33.2
TRIPLICATE 100 copies/10 ^5^ CELLS	36.4	36.6	36.9
TRIPLICATE 10 copies/10 ^5^ CELLS	38	38.7	38.6
CMV **LOD =10** copies/10 ^5^ CELLS, **CT value ranged (30-39)± 3**			

**Analytical-sensitivity**

The ability of the In-house designed multiplex qPCR assay for detection of CMV and EBV DNA simultaneously in the same sample was evaluated through the mixing of two single positive samples for viruses (EBV and CMV) in four replicates, each replicate contain same viral load for both viruses ( Table 4).

**Table 4.** Analytical sensitivity for simultaneous detection.

CMV * copies/10 ^5^cells	EBV ** copies/10 ^5^cells	+ CMV	+ EBV
10 ^3^	10 ^3^	4/4	4/4
10 ^2^	10 ^2^	4/4	4/4
10	10	1/4	2/4

* **CMV**=100% sensitivity at 10 ^2^ copies/10 ^5^ cells and at 10 ^1^ copies/10 ^5^ cells, the sensitivity is 25%

** **EBV**=100% sensitivity at 10 ^2^ copies/10 ^5^cells and at 10 ^1^ copies/10 ^5^, sensitivity is 50%.

**Intra- and Inter-assay reproducibility**

The Intra-assay reproducibility of CMV and EBV In-house designed multiplex qPCR kit was determined by testing 4 replicates of 2 samples each containing 10 ^3^ copies/10 ^5^ cells, 10 ^2^ copies/10 ^5^ cells, and 10 copies/10 ^5^ cells. The CV% was found to be (1.9-0.7%) for EBV, the CT value ranged (34-39), and the CV% was (1.2-0.9%); for CMV, the CT value ranged (33.5-38), while the Inter-assay variability was determined by testing 4 replicates of 2 samples in 2 runs on two different machines each containing 10 ^3^ copies/10 ^5^ cells, 10 ^2^ copies/10 ^5^ cells, 10 copies/10 ^5^ cells, CV% was (1.2-1.3%) for EBV and (1.16-1.36%) for CMV.

**The In-house designed multiplex qPCR assay cross-reactivity**

The specificity or cross –reactivity determined first by NCBI Nucleotide BLAST software ( http://blast.ncbi.nlm.nih.gov/) demonstrated that the primers do not attach to any other sequences except for specific targets. Second, the human DNA was used to check the non-specific binding to human DNA; in addition, 15 negative samples for each virus were tested. The findings revealed 100% specificity.

### 3- In-house designed multiplex qPCR kit vs commercial kit

**Figure 5.**
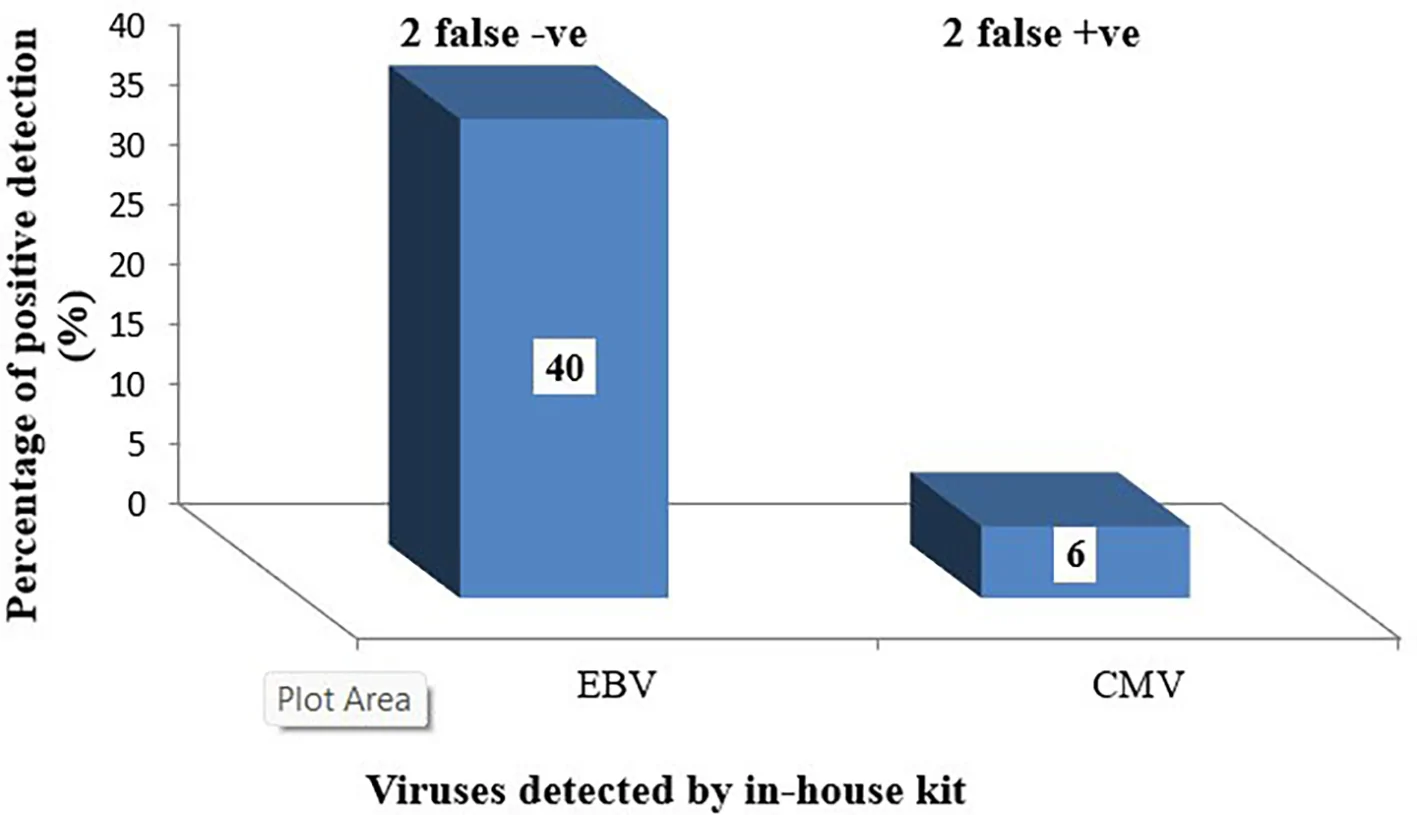
Percentage of EBV and CMV positive MP10 whole blood samples from blood donors detected using the In-house multiplex PCR assay. The In-house kit identified 40% EBV-positive and 6% CMV-positive samples. Comparison with the commercial kit revealed two false-negative EBV results and two false-positive CMV results, indicating minor discrepancies in detection performance.

The 100 mini-pooled whole blood samples from blood donors were retested using the In-house-designed multiplex qPCR. The results revealed that 40% of the mini-pool samples were positive for EBV, while 6% were positive for CMV. The specificity and sensitivity of the In-house-designed multiplex qPCR were calculated by comparison with the Sacace multiplex kit (commercial kit) results, which were considered the gold standard (dependable reference method) to determine the true positive, true negative, false positive, and false negative samples, as shown in Figure 5. Accordingly, the sensitivity and specificity of the In-house-designed kit for CMV detection in MP10 whole blood samples of blood donors were 100% (95% CI: 39.76–100.00) and 97.92% (95% CI: 92.68–99.75), respectively. In contrast, the sensitivity and specificity for EBV detection by the In-house-designed multiplex qPCR in MP10 whole blood samples were 95.24% (95% CI: 83.84–99.42) and 100% (95% CI: 93.84–100.00), respectively.

## Discussion

The use of seronegative blood donors as well as leukoreduced blood remains the safest approach to reduce the incidence of transfusion-transmitted CMV (TT-CMV) and EBV infections among at-risk individuals ( Trottier *et al.*, 2016; Ziemann & Hennig, 2014). However, the risk of CMV infection during the window period may increase the likelihood of TT-CMV among high-risk recipients. Notably, approximately 80–90% of blood donors are CMV-seropositive ( Ziemann & Hennig, 2014), and EBV can still be detected in leukoreduced products ( Trottier *et al.*, 2016). Therefore, obtaining sufficient CMV-seronegative blood donors may be problematic ( Adane & Getawa, 2021). In light of these findings, many researchers have focused on the detection of CMV DNA and EBV DNA in whole blood or plasma from blood donors using PCR to develop suitable infection-prevention strategies ( Eslami Kojidi *et al.*, 2024; Bevan *et al.*, 1991).

The current study revealed 4% and 6% CMV-positive mini-pools by NAT using the commercial and In-house-designed multiplex PCR assays, respectively, while 42% and 40% of mini-pools were EBV-positive by the commercial and In-house assays, respectively. Within each positive EBV mini-pool, two to three individual samples per ten were EBV-positive, whereas one individual sample was CMV-positive. Yushan Xu *et al*. reported 4.15% CMV DNA positivity in 820 whole-blood samples and 1.83% EBV DNA positivity samples using multiplex real-time PCR in 2024 ( Xu *et al.*, 2024). Compared with these findings, the present results indicate a relatively high prevalence of EBV DNA and a moderate level of CMV DNA among Iraqi blood donors, which may represent a real-life threat for high-risk blood transfusion recipients.

These findings are consistent with observations by Lazzarotto *et al*. (2020), who reported that CMV DNA load values in whole blood were consistently higher than in plasma during early infection until reaching the viral load peak, after which they decreased more rapidly in plasma ( Lazzarotto *et al.*, 2020). Similarly, Hudnall *et al*. (2008) quantified all eight human herpesviruses in blood leukocytes from 100 randomly selected donors in southeast Texas using real-time PCR; they reported EBV and CMV positivity rates of 72% and 1%, respectively ( Hudnall *et al.*, 2008). The difference may relate to CMV’s median doubling time of 4.3 days, which is considerably longer than EBV’s doubling time of 46–56 hours ( Cromer *et al.*, 2013; Lodding *et al.*, 2015; Sica *et al.*, 2009).

In this study, whole-blood samples were used rather than serum or plasma based on the premise that EBV and CMV establish latent infections in B lymphocytes and monocytes/macrophages, respectively. This assumption aligns with the findings of Kraft *et al*. ( Kraft *et al.*, 2012), who demonstrated that CMV DNA detection and viral load values are higher in whole blood. Likewise, Rzepka *et al*. (2023) concluded that EBV DNA detection is more sensitive in whole-blood samples than in plasma ( Rzepka *et al.*, 2023).

The multiplex qPCR assay employed in this study enabled the simultaneous detection of EBV and CMV DNA in blood donors, offering insight into the potential of this method to improve blood-screening efficiency in Iraqi blood banks by simplifying workflow, reducing turnaround time, and minimizing cost. This finding corresponds with that of Damian *et al*. (2024), who demonstrated that multiplex qPCR is a cost-effective and robust technique for viral monitoring in kidney transplant recipients ( Damian *et al.*, 2024). Accordingly, the development of an In-house-designed multiplex qPCR kit and its comparison with a commercial assay revealed promising results, particularly when combined with the mini-pool testing strategy, which significantly reduces time and cost while maintaining high sensitivity and specificity.

This concept has been successfully implemented previously by Markoulatos *et al.* (2001) and more recently by Luzius *et al*. (2025), who utilized sensitive multiplex PCR assays capable of simultaneously detecting herpes simplex virus types 1 and 2, varicella-zoster virus, CMV, and EBV in cerebrospinal fluid (CSF) specimens with high accuracy ( Luzius *et al.*, 2025; Markoulatos *et al.*, 2001). The In-house-designed multiplex qPCR assay in the present study produced encouraging results when compared to the commercial reference assay in terms of specificity and sensitivity.

Nevertheless, some limitations were encountered. Firstly, the number of positive (fifteen) and negative controls used for validation of the In-house assay was limited, as it was challenging to obtain newly infected patients for both viruses a stage when viral loads are typically at their peak. Hence, serological tests (IgM+) and nucleic acid testing were used to identify recent or reactivated infections, with the highest viral load observed being 1000 copies per 10 ^5^ cells in the positive group. Secondly, cross-reactivity testing was conducted against human DNA using negative control samples and confirmed via the NCBI Nucleotide BLAST software ( http://blast.ncbi.nlm.nih.gov/), while bacterial DNA cross-reactivity was assessed only in silico without experimental verification. Despite these limitations, both the In-house and commercial multiplex qPCR assays were applied for the first time in Iraq for blood donor screening of CMV and EBV DNA, representing a promising step toward improving blood safety for high-risk patient populations.

## Conclusion

Taken together, the rate of EBV in whole blood was high as compare with moderately low level of CMV in Iraqi blood donors. To eliminate this health problem the using of NAT is the best choice complementary to serological tests, but implementation of NAT is cost, need highly lab requirements with professional trained staff. The In-house designed multiplex qPCR kit when applied on 10 minipooled samples succeeded in amplifying 3 targets (CMV, EBV, and IC) at the same amplification reaction which correspondingly reduce the cost, time and was confirmed to be highly sensitive, specific with reproducible results compared to commercially dependable multiplex qPCR kit.

## Data Availability

The data relating to the findings of this study are available at zenodo, In-house designed Multiplex Real-Time PCR Assay Developed for Epstein - Barr virus and Cytomegalovirus DNA detection in Pooled Blood Samples from Iraqi blood donors. DOI: (10.5281/zenodo.18342023); https://doi.org/10.5281/zenodo.18342023 [ Majid *et al.*, 2026]. Data are available under the terms of Creative Common Attributes 4.0 International License (CC-BY-4.0).
