# Supplementary material for: In-house designed Multiplex Real-Time PCR Assay Developed for Epstein - Barr virus and Cytomegalovirus DNA detection in Pooled Blood Samples from Iraqi blood donors
Source: F1000Res. 2026 Jun 25;15:360. Originally published 2026 Mar 5. [Version 2] doi: 10.12688/f1000research.173833.2 (PMC13550905; doi:10.12688/f1000research.173833.2)
Supplement: Peer review history

# Reviewer report: Reviewer response for version 2

**DOI:** 10.5256/f1000research.201804.r496644

**Published:** 2026-07-07

**Authors:** Noor. Hameed Hanoush

**Affiliations:** 1 University of Anbar, Ramadi, Iraq

I have no other comments. The authors have satisfactorily addressed all of my previous comments.

Is the work clearly and accurately presented and does it cite the current literature?

Yes

If applicable, is the statistical analysis and its interpretation appropriate?

I cannot comment. A qualified statistician is required.

Are all the source data underlying the results available to ensure full reproducibility?

Yes

Is the study design appropriate and is the work technically sound?

Yes

Are the conclusions drawn adequately supported by the results?

Partly

Are sufficient details of methods and analysis provided to allow replication by others?

Yes

Reviewer Expertise:

My area expertise immunogenetics and haematological disease

I confirm that I have read this submission and believe that I have an appropriate level of expertise to confirm that it is of an acceptable scientific standard.

# Reviewer report: Reviewer response for version 2

**DOI:** 10.5256/f1000research.201804.r496645

**Published:** 2026-06-29

**Authors:** xiao zhang

**Affiliations:** 1 Chongqing Medical University, Chongqing, China

No more comments for the authors

Is the work clearly and accurately presented and does it cite the current literature?

Yes

If applicable, is the statistical analysis and its interpretation appropriate?

Yes

Are all the source data underlying the results available to ensure full reproducibility?

Yes

Is the study design appropriate and is the work technically sound?

Partly

Are the conclusions drawn adequately supported by the results?

Partly

Are sufficient details of methods and analysis provided to allow replication by others?

Partly

Reviewer Expertise:

please see my comments

I confirm that I have read this submission and believe that I have an appropriate level of expertise to confirm that it is of an acceptable scientific standard.

# Reviewer report: Reviewer response for version 1

**DOI:** 10.5256/f1000research.191684.r481703

**Published:** 2026-05-19

**Authors:** Noor. Hameed Hanoush

**Affiliations:** 1 University of Anbar, Ramadi, Iraq

The study support the reliability and diagnostic performance of the in-house designed multiplex qPCR assay for simultaneous detection of Cytomegalovirus (CMV) and Epstein–Barr virus (EBV) DNA. 

 The analytical sensitivity of the assay was evaluated using mixed positive samples containing both viral targets in multiple replicates, confirming the assay’s capability to detect CMV and EBV simultaneously within the same specimen

 The in-house multiplex qPCR assay was compared with a commercially available reference kit manufactured by Sacace Biotechnologies, which was considered the gold standard method. Additionally, the development of an in-house assay may provide economic advantages and improve accessibility in resource-limited laboratory settings.

 Despite these strengths, several limitations should be considered:

 The sample size was relatively limited, as only 100 mini-pooled blood donor samples were included, which may restrict the generalizability of the findings. 

  The study did not extensively evaluate cross-reactivity with other clinically relevant herpesviruses, such as Herpes Simplex Virus which could further strengthen the specificity assessment. The use of pooled samples may also have reduced the viral concentration in low-titer specimens, potentially affecting assay sensitivity.

Is the work clearly and accurately presented and does it cite the current literature?

Yes

If applicable, is the statistical analysis and its interpretation appropriate?

I cannot comment. A qualified statistician is required.

Are all the source data underlying the results available to ensure full reproducibility?

Yes

Is the study design appropriate and is the work technically sound?

Yes

Are the conclusions drawn adequately supported by the results?

Partly

Are sufficient details of methods and analysis provided to allow replication by others?

Yes

Reviewer Expertise:

My area expertise immunogenetics and haematological disease

I confirm that I have read this submission and believe that I have an appropriate level of expertise to confirm that it is of an acceptable scientific standard, however I have significant reservations, as outlined above.

# Response

**Published:** 2026-05-28

**Authors:** Ghinwa Majid

**Affiliations:** biology, Al-Nahrain univercity/college of sciences, Baghdad, lecturer, Iraq

**We would like to greatly thanks Editor and Reviewers for their valuable comments and suggestions regarding our manuscript. We highly appreciate the time and effort invested in reviewing our work. The comments were carefully considered. Detailed responses to each comment are provided below.**

**Response 1:**

 Thank you for your valuable observation. The study initially included **1000 whole blood donor samples**, which were subsequently combined into **100 mini-pools** (10 samples per pool). In addition, the study included control groups consisting of **15 CMV-positive samples, 15 CMV-negative samples, 15 EBV-positive samples, and 15 EBV-negative samples**.

 Furthermore, the seroprevalence of both **EBV** and **CMV** is relatively high in Iraq, which increases the likelihood of detecting these viruses even when using a sample size of fewer than 100 pooled samples. Therefore, we believe that the sample size used in this study was adequate to achieve the study objectives and provide meaningful preliminary findings.

**Response2:**

 Thank you for this important observation regarding cross-reactivity. The possibility of cross-reactivity was already mentioned in the limitations section of the discussion. In the present study, cross-reactivity with bacterial pathogens was assessed in silico, while cross-reactivity with human DNA was evaluated both experimentally and in silico to avoid false-negative results and ensure assay specificity.

 Regarding cross-reactivity with other pathogens, including viruses and bacteria, even if limited cross-reactivity occurs, this may still be considered beneficial in the context of blood transfusion safety. Detection of potentially contaminated blood units, regardless of the exact pathogen, could help prevent transfusion of infected blood to high-risk or immunocompromised patients. Therefore, such findings may contribute positively to precautionary blood donor screening and transfusion safety measures.

**Response 3:**

 Thank you for this important comment regarding the possible dilution effect associated with pooled samples. The potential reduction in viral concentration after pooling was carefully evaluated during the preliminary phase of the study by testing pools containing 5, 10, and 15 whole blood samples. Based on the obtained findings, pooling of 10 samples per pool was selected as the most appropriate strategy, as it provided acceptable assay sensitivity and performance while achieving the main objective of the study, which was to reduce the overall cost of molecular screening.

 Moreover, sample pooling is widely used strategy especially in surveillance of large numbers of samples like in blood transfusion samples, particularly where the number of samples exceeded thousands, therefore the pooling system a good choice to reduce the time , cost   and blood transfusion safety.

# Reviewer report: Reviewer response for version 1

**DOI:** 10.5256/f1000research.191684.r469726

**Published:** 2026-04-07

**Authors:** xiao zhang

**Affiliations:** 1 Chongqing Medical University, Chongqing, China

This study established an in-house multiplex real-time PCR assay for EBV and CMV detection in Iraqi blood donors, which is of important clinical significance for ensuring blood transfusion safety. However, critical issues in methodological validation, sample quality control, and reference formatting require revision to improve the scientific rigor of the manuscript.

**Major Comments:**

 1.The in-house developed kit was validated using only 15 positive and 15 negative samples. Such an insufficient validation sample size makes it difficult to comprehensively evaluate the stability, repeatability and clinical generalizability of the detection method, and may easily lead to biased results in the assessment of sensitivity and specificity.

 2. The blood donor samples used in this study were collected from 2018 to 2019 with an excessively long storage period. The authors did not specify the DNA storage conditions or nucleic acid degradation quality control data, so the interference of aged samples on viral nucleic acid detection results cannot be excluded, which affects the authenticity and reliability of the research findings.

 3. Insufficient sample size for validation. Only 15 positive and 15 negative controls were used to evaluate the performance of the in-house assay, resulting in wide confidence intervals and limited statistical power.

 4. Potential dilution effect from the pooling strategy. The 10:1 pooling strategy (MP10) may lead to false negatives due to dilution of low-copy-number samples. The authors should address this issue experimentally or thoroughly discuss this risk in the manuscript.

 5. Lack of independent validation of the commercial kit used as the gold standard. The study used the commercial Sacace kit as the reference method, but the manuscript does not provide its performance characteristics (sensitivity, specificity, limit of detection).

**Minor Comments:**

 1. The reference list has inconsistent formatting in the presentation of volume, issue and page numbers, punctuation usage, and supplementary link annotations. These items are not standardized and should be revised uniformly in accordance with the journal’s citation requirements.

 2. Typographical error in PCR cycling conditions. In the "DNA amplification" section, the authors wrote "40 mins at 63℃," which is likely a mistake. The annealing/extension step in real-time PCR typically lasts seconds, not minutes. Please correct to "40 seconds" or confirm the actual protocol.

Is the work clearly and accurately presented and does it cite the current literature?

Yes

If applicable, is the statistical analysis and its interpretation appropriate?

Yes

Are all the source data underlying the results available to ensure full reproducibility?

Yes

Is the study design appropriate and is the work technically sound?

Partly

Are the conclusions drawn adequately supported by the results?

Partly

Are sufficient details of methods and analysis provided to allow replication by others?

Partly

Reviewer Expertise:

please see my comments

I confirm that I have read this submission and believe that I have an appropriate level of expertise to confirm that it is of an acceptable scientific standard, however I have significant reservations, as outlined above.

# Response

**Published:** 2026-05-15

**Authors:** Ghinwa Majid

**Affiliations:** biology, Al-Nahrain univercity/college of sciences, Baghdad, lecturer, Iraq

**We would like to express our sincere gratitude to the Editor and Reviewers for their valuable comments and constructive suggestions regarding our manuscript. We highly appreciate the time and effort invested in reviewing our work. The comments were carefully considered, and the manuscript has been revised accordingly to improve its scientific quality and clarity. Detailed responses to each comment are provided below.**

**Major Comments:**

**Response to Comment 1**

 This limitation has been acknowledged and clearly stated in the discussion section of the revised manuscript. Although the initial validation included 15 positive and 15 negative individual samples, the in-house designed kit was additionally evaluated using 100 pooled clinical samples.

 These pooled samples were first tested using the commercially approved Sacace kit as a reference method and were subsequently retested using the in-house developed kit. The comparative analysis demonstrated consistent detection performance between both methods, supporting the preliminary reliability of the developed assay.

**Response to Comment 2**

 All samples were stored frozen at −80 °C in accordance with the manufacturer’s instructions until processing and molecular analysis were performed.

 We appreciate the reviewer’s observation, and this information has now been clearly added to the sample collection section in the newly revised manuscript.

**Response to Comment 3:**

 We acknowledge that the number of positive and negative samples used for the initial validation of the in-house assay was relatively limited, which may affect the statistical power and confidence intervals of the obtained results. However, the primary objective of this study was to perform a preliminary evaluation of the assay performance under controlled laboratory conditions. In addition to the 15 positive and 15 negative control samples, the in-house assay was further evaluated using 100 pooled clinical samples that were initially tested with the approved Sacace reference kit and subsequently retested using the developed assay. The obtained results demonstrated comparable detection performance between both kits **.**

**Response to Comment** **4**:

 We thank the reviewer for this important comment and for highlighting the need to clarify the pooling validation process in the manuscript.

 In addition, pooling validation was conducted using 10 whole blood samples per pool after preliminary evaluation of pools containing 5 and 15 samples. Based on the obtained results, pooling of 10 samples was selected as the most appropriate strategy, as it provided acceptable assay performance while supporting the main objective of the study, which was to reduce the overall cost of molecular screening.

 This clarification regarding the pooling validation strategy has now been added to the revised manuscript.

**Response to Minor Comments**

**1- **We have carefully revised the reference list to ensure consistency and standardization in formatting, including uniform presentation of volume, issue, page numbers, punctuation, and supplementary link annotations, in accordance with the journal’s citation style requirements **.**

**2- **We appreciate the reviewer’s observation regarding the PCR cycling conditions. The typographical error in the “DNA amplification” section has been corrected, and the annealing/extension step has been revised from “40 mins at 63℃” to “40 seconds at 63℃” to accurately reflect the actual protocol used.

 These corrections have been implemented in the revised manuscript
